# Automated larval motility assays reveal links between eprinomectin treatment failure and drug resistance in *Haemonchus contortus*

**DOI:** 10.1186/s13567-025-01622-9

**Published:** 2025-10-02

**Authors:** Julie Petermann, Marie Garcia, Fabrice Guegnard, Christelle Grisez, Sophie Jouffroy, Léa Bordes, Cédric Neveu, Mickaël Riou, Guillaume Sallé, Philippe Jacquiet, Mélanie Alberich, Anne Lespine

**Affiliations:** 1 IHAP, Université de Toulouse, INRAE, ENVT, Toulouse, France; 2 INTHERES, Université de Toulouse, INRAE, ENVT, Toulouse, France; 3 ISP, Université de Tours, INRAE, 37380 Nouzilly, France; 4 UE-1277 Plate-Forme d’Infectiologie Expérimentale (PFIE), INRAE, Nouzilly, France

**Keywords:** *Haemonchus contortus*, phenotyping, eprinomectin resistance, dairy sheep, larval motility

## Abstract

Small ruminants are frequently infected with gastrointestinal nematode parasites (GIN) such as the highly pathogenic *Haemonchus contortus*, which severely impact animal health, welfare and production performance. The increasing prevalence of clinical anthelmintic resistance poses a global threat to effective parasite control and the productivity of livestock farming. This includes resistance to eprinomectin (EPR), the only anthelmintic currently approved for use in dairy production with a zero-withdrawal period. This study aims to link EPR therapeutic failure against *H. contortus* in dairy sheep farms in southwestern France with drug potency, as determined via the larval motility phenotype in an in vitro test. Six field isolates (four EPR-resistant and two EPR-susceptible isolates) were collected from dairy sheep farms, where EPR efficacy was assessed using the faecal egg count reduction test (FECRT). In addition, two laboratory isolates, known for their EPR-susceptible status, were used. We simultaneously evaluated the effects of ivermectin (IVM), moxidectin (MOX), EPR, and levamisole on larval stage development and motility, comparing putative EPR-resistant isolates with those expected to be EPR-susceptible. The automated motility assay effectively distinguished EPR-susceptible isolates from EPR-resistant isolates, with IC_50_ values between 0.29 and 0.48 µM for susceptible isolates and between 8.16 and 32.03 µM for resistant isolates, revealing that isolates from farms with EPR treatment failure presented high resistance factors for EPR, ranging from 17 to 101. Our results reveal the sensitivity, reliability, and reproducibility of the motility test in monitoring the response of *H. contortus* to eprinomectin, and it may be used to detect *H. contortus* resistance to eprinomectin on farms. This paves the way for improving diagnostics and treatments for helminth infections in dairy sheep farms.

**Supplementary Information:**

The online version contains supplementary material available at 10.1186/s13567-025-01622-9.

## Introduction

Anthelmintic resistance has emerged as a critical global crisis, posing a severe threat to effective parasite control and the productivity of livestock farming. The overuse of anthelmintics such as benzimidazoles and ivermectin (IVM) over decades has set the stage for this crisis, but more recently, the alarming spread of clinical resistance to eprinomectin (EPR) in southwestern France has intensified concerns [1]. This region is not only a major hub for French dairy sheep production but also the heartland of milk production for Protected Designation of Origin (PDO) cheeses. The rapid increase in the prevalence of resistance in this vital sector threatens both the sustainability of farming practices and the economic viability of PDO cheese production, highlighting the urgent need for new strategies and solutions to combat this growing menace.

The PDO specifications require long periods of grazing during the year, which exposes ewes to gastrointestinal nematode parasites (GIN), such as the highly pathogenic *Haemonchus contortus,* whose infections alter animal health, welfare and production performance. This species thrives under tropical conditions but has been increasingly encountered in southwestern French regions [2, 3], where climatic conditions are highly suitable for larval development. Treatment predominantly relies on anthelmintics, including EPR, which is the most commonly used anthelmintic in dairy sheep and has become critical for controlling GIN on these farms. Importantly, the EPR is the only anthelmintic authorized in France with a zero-withdrawal period for milk, allowing its use throughout the ewes'lactation period, which lasts approximately eight months per year. The first documented case of reduced clinical effectiveness of EPR against *H. contortus* in a goat farm in the Pyrénées-Atlantiques department was described in 2019 [3]. Since then, an increasing number of similar cases have been reported on dairy sheep farms within the same area [1, 4]. In each case, *H. contortus* was identified as the sole resistant species [1, 3].

In that respect, there is an urgent need for rapid and reliable methods to detect and monitor reduced susceptibility to anthelmintics in field parasites. Reduced susceptibility refers to parasites that have developed resistance mechanisms, making them phenotypically distinct from the wild population and enabling early adaptation to treatment strategies [5, 6]. The clinical efficacy of anthelmintics is usually assessed in farms using faecal egg count reduction tests (FECR) following WAAVP guidelines [7, 8] or through simplified composite analysis [9]. However, FECRT is both time-consuming and costly and is generally conducted after clinical signs of drug failure have been observed [10–12]. In addition, inaccuracies in faecal egg counts and the low sensitivity of the FECRT often result in late-stage resistance diagnosis [5, 12].

In vitro diagnostic of drug resistance tests using the free-living stages of parasites (eggs or L3) provide a reliable alternative [5, 6, 12] but with some constraints. For example, the egg hatch assay and genetic markers are limited to benzimidazoles, and the egg hatch assay requires rapid and anaerobic shipping to avoid premature egg development [13]. The larval development assay (LDA) allows the measurement of the capacity of drugs to block the development of nematodes from eggs to L3. This test was developed primarily for *H. contortus* and *T. colubriformis* [14, 15], and it is currently the most widely used commercially available test for the detection of anthelmintic resistance to benzimidazoles, imidazothiazoles and macrocyclic lactones (ML) [16]. LDA has practical limitations, as it requires fresh faeces sent quickly and vacuum-sealed to prevent premature development of the eggs [16], inflicting considerable logistical constraints. Additionally, its sensitivity in detecting resistance to moxidectin is limited [6]. The development of easier and more affordable diagnostic tools would facilitate epidemiological surveys, help identify risk factors for resistance [4, 17, 18], and open the way for effective nematode control while preventing the spread of anthelmintic-resistant GIN.

Tests assessing larval motility and mobility in the presence of increasing concentrations of anthelminthics can distinguish susceptible from resistant larvae [11, 19–21]. The automatization of motility or mobility measurements makes them less subjective and time-consuming and more sensitive and repeatable. Recently, an automated WMicroTracker™ One apparatus was applied as a fast and reliable functional indicator of nematode motility to monitor the response to ML and to detect resistance in adult *Cenorhabditis elegans* and *H. contortus* L3 larvae [22]. Concurrently, we identified a cluster of dairy sheep farms in southwestern France where *H. contortus* infections were unresponsive to EPR, as assessed by the FECRT. Failure of drug administration was ruled out by verifying that the serum drug concentration in the treated sheep was sufficient to ensure anthelmintic efficacy, suggesting that the worms had developed resistance to eprinomectin [1].

This study aims to link the therapeutic failure of EPR against *H. contortus* on dairy sheep farms in southwestern France with the larval motility phenotype in an in vitro test. To achieve this goal, we used the WMicroTracker™ One automated methodology to assess the impact of the drug on *H. contortus* motility and evaluated its predictive value compared with that of the conventional LDA and FECRT. We concurrently monitored the effects of ivermectin (IVM), moxidectin (MOX), EPR, and levamisole (LEV) on development and motility by comparing putative ML-resistant worms with isolates expected to be drug susceptible.

## Materials and methods

This work was performed on eight isolates. Four isolates were EPR-susceptible, including two reference isolates and two field isolates. The other four were EPR-resistant field isolates.

### Two reference isolates

To carry out this work, we used two reference drug-susceptible isolates: the Weybridge isolate, which was isolated in the United Kingdom before 1980 [23], and the Humeau isolate, which was isolated in France in Gers County before 2000 [24]. Both isolates are susceptible to ML and have never been exposed to synthetic EPR. They have been maintained in the laboratory since their isolation. The field isolates presented here will be compared with these two reference isolates.

### Collection of field isolates

#### Farm selection

Six dairy sheep farms located in the Pyrénées-Atlantiques *département* (France) were visited in 2020 and 2021 on the basis of farmers’ or veterinarians’ demands and suspicions of EPR unresponsiveness. All the farmers produced milk for the Protected Designation of Origin (PDO) Ossau Iraty cheese; hence, the ewes grazed for at least 240 days during the year.

#### FECR after EPR treatment on farms

The efficacy of EPR treatment was evaluated using the FECRT according to the WAAVP recommendations [7, 25]. Ewes of Basco-bearnaise or Red Face Manech breeds, depending on the farm, were randomly assigned to the treatment group and were identified accordingly. The treatment group consisted of 10 or 11 animals that received a subcutaneous injection of 0.2 mg/kg eprinomectin (Eprecis^®^ injectable, CEVA Santé Animale, Libourne, France). All the animals were treated at a dose corresponding to a body weight (BW) of 80 kg, which is greater than that of the heaviest animal in the group. Faeces from all the animals were collected individually on the treatment day and fourteen days later. The samples were duplicated for the FECRT and for larval culture and identified via the 5-digit number tags of the animals.

Strongyle egg counts in faeces were carried out via the McMaster method modified by Raynaud, which uses 3 g of faeces per animal. The sensitivity of this method is 15 eggs per gram [26]. The reduction in the number of eggs excreted in the faeces after treatment was calculated according to the [25] formula:

$$FECRT=100\times \left(1-\frac{{m}_{t2}}{{m}_{t1}}\right),$$

where m_t1_ and m_t2_ are the arithmetic means of FEC in a treated group at D0 and D14.

The confidence intervals (CI) were calculated according to [27] via the following formula:

$$CI = 100\left[1-\left(\frac{{m}_{t2}}{{m}_{t1}}\right)\text{exp}\left(\pm t\sqrt{V}\right)\right]$$

$$V=\frac{{S}_{t2}^{2}}{{n}_{t}{m}_{t2}^{2}}+\frac{{S}_{t1}^{2}}{{n}_{t}{m}_{t1}^{2}}-2\times \frac{cov\left(t1t2\right)}{{n}_{t}{m}_{t1}{m}_{t2}},$$

where ${n}_{t}$ is the number of animals included in the test, ${S}_{t1 }^{2}$ and ${S}_{t2}^{2}$ are the variances of FEC at D0 and D14, respectively, and *cov(t1t2)* is the covariance estimate between the pre- and postdrenching analyses.

The interpretation of the FECRT results was made following COMBAR 2021 recommendations [28].

Calculation of the reduction percentage was also performed on the basis of revised WAAVP recommendations [8]. Individual data on faecal egg counts before and after treatment were analysed via the web application [29].

### Preparation of larvae of field isolates

#### Susceptible field isolates

Two of the field isolates collected from farms where EPR treatment was effective contained multiple parasite species. To specifically select *H. contortus* worms, a preliminary selection stage was conducted by experimentally infecting two 3- to 4-month-old lambs per field isolate. To ensure that the lambs were free of any preexisting parasitic infections, they were treated 10 days before infection with Fenbendazole (Panacur^®^, INTERVET, 10 mg/kg of BW) and Levamisole (Biamine 5%^®^, LABORATOIRE BIARD, 7.5 mg/kg of BW) to eliminate any existing gastrointestinal nematodes and with diclazuril (Vecoxan^®^, INTERVET, 1 mg/kg of BW) to prevent coccidiosis.

Lambs were then infected with 6000 L3 and slaughtered 35 days post-infection in strict accordance with the national ethical guidelines. The abomasum was removed and stored at 39 °C and then opened lengthwise, and the live *H. contortus* worms were collected with a needle. The isolated parasites were washed three times with warm PBS containing 200 U/mL penicillin and 200 µg/mL streptomycin. The final washing before culture was conducted using warm DMEM (Gibco, Thermo Fisher Scientific, Waltham, MA, USA) culture medium at 39 °C supplemented with the same antibiotics. A minimum of 200 adult parasites per isolate were maintained in a total volume of 20 mL in a 25 cm^2^ cell culture flask. The flask was incubated at 39 °C in 5% CO_2_ and humidified air for up to 48 h. Eggs were collected twice daily and allowed to develop under conditions similar to those of LDA. After 10 days, the L3 larvae were collected and stored at 8 °C for a minimum of 15 days prior to infection at 6000 L3 per lamb.

The susceptible field isolates were named Ch and Lu.

#### Resistant isolates

On four farms, the larval population obtained 14 days after EPR treatment was composed of *H. contortus* only on the basis of morphological and molecular analysis [1]. Pure EPR-resistant *H. contortus* isolates were subsequently obtained by infecting lambs with 10 000 larvae of each isolate. Twenty-one days later, the sheep received a subcutaneous injection of 0.2 mg/kg eprinomectin (Eprecis^®^ injectable, CEVA Santé Animale). From day 31 post infection, faecal samples were collected for both the LDA and the production of L3 larvae via faecal culture. The resistant isolates were named Ar, Be, Bu and Mo.

#### Larval amplification for motility assay

All tested isolates were amplified from sheep as follows. Prior to infection, the sheep were treated for at least 14 days with either monepantel at 2.5 mg/kg (ZOLVIX^®^ 25 mg/mL, ELANCO, 2.5 mg/kg of BW) or levamisole (LEVAMISOLE 3.75% oral drench^®^, HUVEPHARMA, 7.5 mg/kg of BW, stop dose at 0.375 g) to eliminate eventual GIN, and the absence of egg excretion was controlled using faecal egg count (FEC). The sheep were then infected with 10000 larvae for each isolate. Twenty-one days later, the FEC were monitored, and the faeces were used for faecal culture as described below. L3 larvae were kept at 8 °C for a maximum of 5 months.

#### Coprocultures

For the faecal cultures, the faeces were maintained at 100% humidity at 24 (± 1) °C for 12 days to favour egg development into third-stage larvae (L3). After 12 days, the pots containing faeces were fully filled with tap water and were turned upside down in Pétri dishes, which were also filled with tap water. L3 larvae were then collected 24 and 48 h after [30] and stored horizontally in a cell culture flask at 8 °C.

#### Larval development assay (LDA)

The assay was based on the method described by Hubert and Kerboeuf [31]. Twenty-five days after infection of the animals at the PFIE, faecal samples were collected for egg extraction. The collected faeces were mixed with tap water and filtered through two filters of 100 µm and 32 µm. The eggs were collected in a 32 µm filter and mixed with kaolin to pellet them. After centrifugation at 600 × *g* for 5 min, the pellet was resuspended in a saturated NaCl solution at 360 mg/mL. A second centrifugation was performed for 5 min at 900 × *g*. The eggs contained in the supernatant were collected on a 32 µm sieve and thoroughly rinsed with tap water before being collected and counted.

Approximately 100 eggs were placed in a 96-well plate together with food (*E. coli* strain OP50, the strain commonly used to feed larval stages in in vitro conditions [32]), and the treatment was formulated in DMSO. The plates were sealed and incubated at 25 °C for 7 days to allow nematodes to develop to the L3 stage [31, 33]. The proportions of eggs that had evolved to L3 were compared to those in the nontreated well. The ability of LEV, IVM, MOX and EPR to inhibit larval development was measured in a concentration-dependent assay by adding drugs at increasing concentrations (12 concentrations between 0 and 125 μM (0.4 nM, 1.25 nM, 4 nM, 12.5 nM, 40 nM; 0.125 µM, 4 µM, 12.5 µM, 40 µM and 125 µM), in triplicate). All of these compounds were purchased from Sigma‒Aldrich (Saint-Quentin Fallavier, France), solubilized in dimethyl sulfoxide (DMSO) and then diluted in deionized water to a final concentration of 1% in contact with eggs. The final concentration of DMSO used in the assay was less than 1% to exclude any harmful effects of the vehicle.

### Motility assay

#### Chemicals and stock solutions

Levamisole (LEV), ivermectin (IVM), moxidectin (MOX), eprinomectin (EPR), dimethyl sulfoxide (DMSO), bacto agar, bacto peptone, bovine serum albumin (BSA), CaCl2, LB, NaCl and MgSO4 were purchased from Sigma‒Aldrich (St Quentin Fallavier, France). For all the experiments, IVM, MOX and EPR were dissolved in DMSO.

#### Larval motility assay (WMiT)

The motility of *H. contortus* L3s was assessed from score activity recordings via the Worm MicroTracker™ One (WMiT) from PhylumTech (Santa Fe, Argentina), which detects infrared microbeam interruptions due to worm movement in liquid media [22]. When the movement is slowed by exposure to a paralyzing drug, the number of pulses decreases and can reach zero when the worms are completely paralyzed. The method used was adapted from protocols previously described for *H. contortus* [35, 36]. The capacity of LEV, IVM, MOX and EPR to inhibit worm motility was measured in a concentration-dependent assay by adding drugs at increasing concentrations from 0.01 to 100 μM (0.01 µM, 0.1 µM, 0.5 µM, 1 µM, 10 µM, 50 µM and 100 µM). IVM, MOX and EPR were solubilized in DMSO, and the final concentration of DMSO was 0.5%. The negative control consisted of L3 in LB medium. For ML, the negative control medium was supplemented with 0.5% DMSO.

Prior to the experiments, the cuticle of *H. contortus* larvae was removed. Since the L3 cuticle is an important protective factor [16], unsheathing is essential to evenly expose the larvae to the drug [23, 37], and it also reflects the state of potential exposure to drugs in vivo. Most experiments use a 20-min bleaching contact [35, 36] by incubating the larvae at 37 °C in tap water supplemented with 0.15% NaOCl. The incubation duration can vary across experiments [37]. The Weybridge isolate lost its mobility after 20 min of bleaching exposure; hence, the protocol was adapted by reducing the bleach treatment to 10 min. For exsheatment, the larval mixture was vortexed every 5 min. Then, the larvae were filtered through 40 μm mesh in LB medium to eliminate aggregated material. 80 L3 suspensions in 200 μL of LB medium were distributed in each well of a 96-well plate. The plates were subsequently incubated at 37 °C for 24 h in a humidified incubator, maintaining a 5% CO_2_ atmosphere and humidity levels ≥ 90%. Following the incubation period, the larvae were exposed to light at room temperature for at least 5 min to restore their ability to move. The motility of the larvae within each well was then recorded over a 15-min period using WMiT technology. Two infrared microbeams cross each well, and each time they are interrupted, a movement is counted. The motility of the worms in each well was then standardized against the average motility of the control wells to derive the motility inhibition values (%) [22].

For each isolate, the experiments were repeated 2 to 10 times to assess the reproducibility of the results.

### Animal ethical guideline-agreement

*Haemonchus contortus* field isolates were first purified within the Plateforme d’Infectiologie Expérimentale (PFIE, INRAE Centre Val de Loire, Nouzilly, France, 2024 [38]). Eggs, larval stages and adult worms were obtained from experimentally infected lambs under controlled conditions.

Experimental infection, maintenance and euthanasia were conducted in strict accordance with the national ethics guidelines and approved by the local ethics committee for animal experimentation (Comité d’Éthique en Expérimentation Animale Val de Loire, CEEA VdL N°19) under protocol number APAFIS#17,560. The eggs used for the larval development test originated from this infection.

For motility tests with Worm MicroTracker™, a preliminary step of larval amplification was performed on experimentally infected ewes and rams under controlled conditions within the Toulouse National Veterinary School (UMR INRA/ENVT 1225 Interactions Hôtes-Agents Pathogènes (E3155527), approved by the ethics committee for animal experimentation (APAFIS #39,739).

### Data analysis and statistics

For the WMiT test, concentration‒response curves were plotted using the GraphPad Prism 8 software package (GraphPad, San Diego, CA, USA). A normalized linear log-inhibitor model was used to fit the curves and calculate the half-maximal inhibitory concentration (IC_50_) values, which reflect the ability of the drug to achieve the effect. IC_50_ comparisons were performed using an F-test. The resistance factor (RF) was derived as the ratio of the IC_50_ values of the tested and reference isolates:

$$RF=I{C}_{50}\left[tested isolat\right]\div {IC}_{50}[reference isolat]$$

R Studio 4.1.3 was used to calculate the IC_50_ for LDA with the “DRC” [39] package with nonlinear regression LL2.3 (a three-parameter log-logistic function) and to plot the IC_50_ for both the WMiT analysis and the LDA.

## Results

### Collection of field *H. contortus* isolates

We purified and amplified six isolates, four (R-Be, R-Mo, R-Ar, and R-Bu), which were collected from farms with reduced EPR efficacy, as indicated by an FECRT lower than 90%, and the other two (S-Ch and S-Lu), which were collected from farms where the EPR was effective (FECRT > 95%) (Table 1).

**Table 1:** **Origin of *H. contortus*isolates**: *H. contortus* egg*s* were collected and classified as susceptible (S) when issued from farms responsive to the drug or resistant (R) when issued from farms nonresponsive to the EPR.

Isolate	Origin	Collection date	FECRT with EPR treatment [90% CI]	Results from the Delta method (Levecke et al. [47])	Expected phenotype
Weybridge*	UK	Before 1980	non-exposed to ML	Non-exposed to ML	S
Humeau*	Gers (France)	2002	non-exposed to EPR	Non-exposed to EPR	S
Mo	Pyrénées-Atlantiques (France)	June 2020	− 33% [−205%; 42%])	Classification: Resistant 90% CI − 242.1–83.3%	R
Be	Pyrénées-Atlantiques (France)	July 2021	55% [17%; 76%]	Classification: Resistant 90% CI 23.6–79.7%	R
Ar	Pyrénées-Atlantiques (France)	June 2020	83.3% [30%; 96%]	Classification: Resistant 90% CI 54.1–98.5%	R
Bu	Pyrénées-Atlantiques (France)	June 2020	− 19% [−149%; 44%]	Classification: Resistant 90% CI − 146.3–66.7%	R
Ch	Pyrénées-Atlantiques (France)	April 2021	100%	Non-available	S
Lu	Pyrénées-Atlantiques (France)	April 2021	100%	Non-available	S

The classification of the farms is based on the FECRT values calculated according to the WAAVP method [7, 26]; the 90% CI were calculated according to [27].

^*^Reference isolates (see “2.1 reference isolates”).

### Results obtained from the larval motility tests

#### Validation of experimental conditions

The principle of the WMiT assay is based on the movement of worms that cut an infrared beam in wells at a given frequency reflecting full activity. The anthelmintic LEV, a worm paralyzing agent, was used as a positive control for the WMiT assay. We observed that LEV immobilized the larvae regardless of the isolate tested, with IC_50_ values ranging between 9 and 50 µM (Additional file 1).

#### ML potency of *H. contortus* isolates from farms

**Figure 1:**
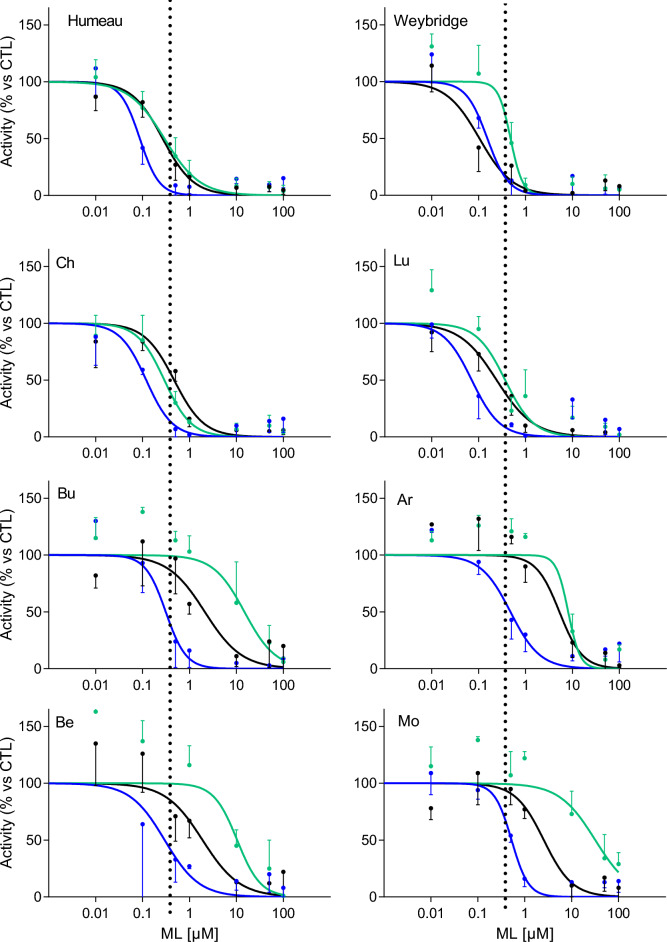
**Concentration‒response curves for MOX (blue), EPR (green) and IVM (black) of the eight** ***H. contortus*** **isolates (Humeau and Weybridge** (reference isolates); **Ch and Lu** (susceptible isolates)**; Bu, Ar, Be and Mo** (Putative EPR-resistant isolates)). MOX: moxidectin; EPR: eprinomectin; IVM: ivermectin; ML: macrocyclic lactones. The black dotted line corresponds to the EPR IC_50_ determined for Humeau. Each graph represents one experiment out of 2–10.

We determined and compared the potencies of three MLs—IVM, MOX, and EPR—on the 4 susceptible isolates (as determined by FECRT for the field isolates) under our experimental conditions. The main finding was that all three MLs altered worm motility, as indicated by these specific test potencies in the µM range across the four drug-susceptible isolates (between 0.09 µM (Lu, IVM) and 0.41 µM (Lu, EPR)) (Figure 1). For the reference strains, Humeau was more susceptible to MOX, with a significantly lower IC_50_ than EPR and IVM (F test, *p* < 0.0001). IVM and EPR had equivalent IC_50_ values (approximately 0.3 µM). The Weybridge strain differed slightly, showing greater susceptibility to IVM and MOX than to EPR (*p* < 0.0005). Additionally, Weybridge bulls were slightly more susceptible to IVM than Humeau bulls were (*p* = 0.0025).

For the two field isolates, Ch and Lu, MOX was the most potent ML (IC_50_ = 0.12 and 0.07 µM, respectively). EPR and IVM exhibited very close potencies that were lower than those for MOX (IC_50_ = 0.29 and 0.47 µM, respectively, for Ch and 0.37 and 0.24 µM, respectively, for Lu) and close to those obtained for the reference strains. Compared with Weybridge, Ch had a slightly lower IC_50_ for EPR (IC_50_ = 0.29 and 0.48 µM, respectively, *p* < 0.05). The IC_50_ values of IVM for Humeau and Lu were similar and lower than that for Ch (*p* < 0.05).

We then determined the potency of IVM, MOX and EPR in altering the motility of the four putative EPR-resistant isolates, as identified by FECRT. Compared with those of Humeau as a reference isolate, a clear shift in the curves to the right for the three drugs was observed for Ar, Bu, Be and Mo (Figure 1), revealing a lower potency of the drugs in these four isolates. This shift was especially notable for EPR, which has a significantly greater IC_50_ (between 8.16 and 32.03 µM for the four resistant isolates) than Humeau (EPR IC_50_ = 0.3 µM, p < 0.0001 for Bu, Be and Mo, Figure 1). These findings suggest that these isolates are highly resistant to EPR. For Ar, we could not statistically assess the difference in the EPR-IC_50_ with Humeau, as the two isolates have different hillslopes. Nevertheless, we also observed a significant increase in the IC_50_ for MOX for the four isolates (between 0.30 and 0.53 µM for the four resistant isolates, *p* < 0.05) and for IVM for the Be (1.9 µM) and Mo (2.56 µM) isolates (*p* < 0.0001). For the Ar and Bu isolates, we could not test the difference in the IC_50_ values, as they have different hillslopes than Humeau.

With respect to the resistance factor (RF) when the Humeau isolate was chosen as a reference, resistant isolates had RFs between 17 and 101 for EPR. Weybridge, Lu and Ch have RFs between 0.7 and 1. For IVM, the RF for resistant isolates ranged from 2.3 to 10.8, whereas the RF for the EPR-susceptible isolates ranged from 0.5 to 1.1. For MOX, the RFs for the resistant isolates ranged from 1.1 to 3.4, whereas the RFs for the susceptible isolates ranged from 0.7 to 1.2 (Table 2). The order of magnitude is quite the same when the Weybridge isolate is chosen as a reference, with, for example, an RF between 24 and 139 for the resistant isolate for EPR (Table 2).

**Table 2:** **IC**_**50**_** values (µM) obtained with WMiT for MOX, EPR and IVM for** ***Haemonchus contortus*** **isolates and Resistant Factors (RF) calculated with the Humeau isolate as a reference.**

IC_50_ (µM)	EPR				IVM				MOX				Number of experiments
Isolates	Mean	SD	RF/Humeau	RF/Weybridge	Mean	SD	RF (Humeau)	RF/Weybridge	Mean	SD	RF (Humeau)	RF/Weybridge	
Humeau	0.40	0.23	–	1.4	0.34	0.18	–	1.5	0.13	0.10	-	0.8	10
Weybridge	0.29	0.26	0.7	–	0.23	0.17	0.7	–	0.16	0.00	1.2	–	4
Ar	18.81	15.07	47	65	3.71	2.25	10.8	16	0.34	0.19	2.6	2	2
Be	6.86	3.75	17	24	0.90	0.90	2.6	4	0.14	0.14	1.1	1	3
Bu	40.40	29.41	101	139	0.77	0.15	2.3	3	0.24	0.10	1.8	1.5	3
Ch	0.33	0.21	0.8	1.1	0.37	0.25	1.1	1.6	0.11	0.06	0.8	0.7	3
Lu	0.41	0.23	1	1.4	0.18	0.15	0.5	0.8	0.09	0.06	0.7	0.6	4
Mo	25.82	8.79	64.5	89	2.05	0.73	6	9	0.44	0.13	3.4	2.8	2

**Figure 2:**
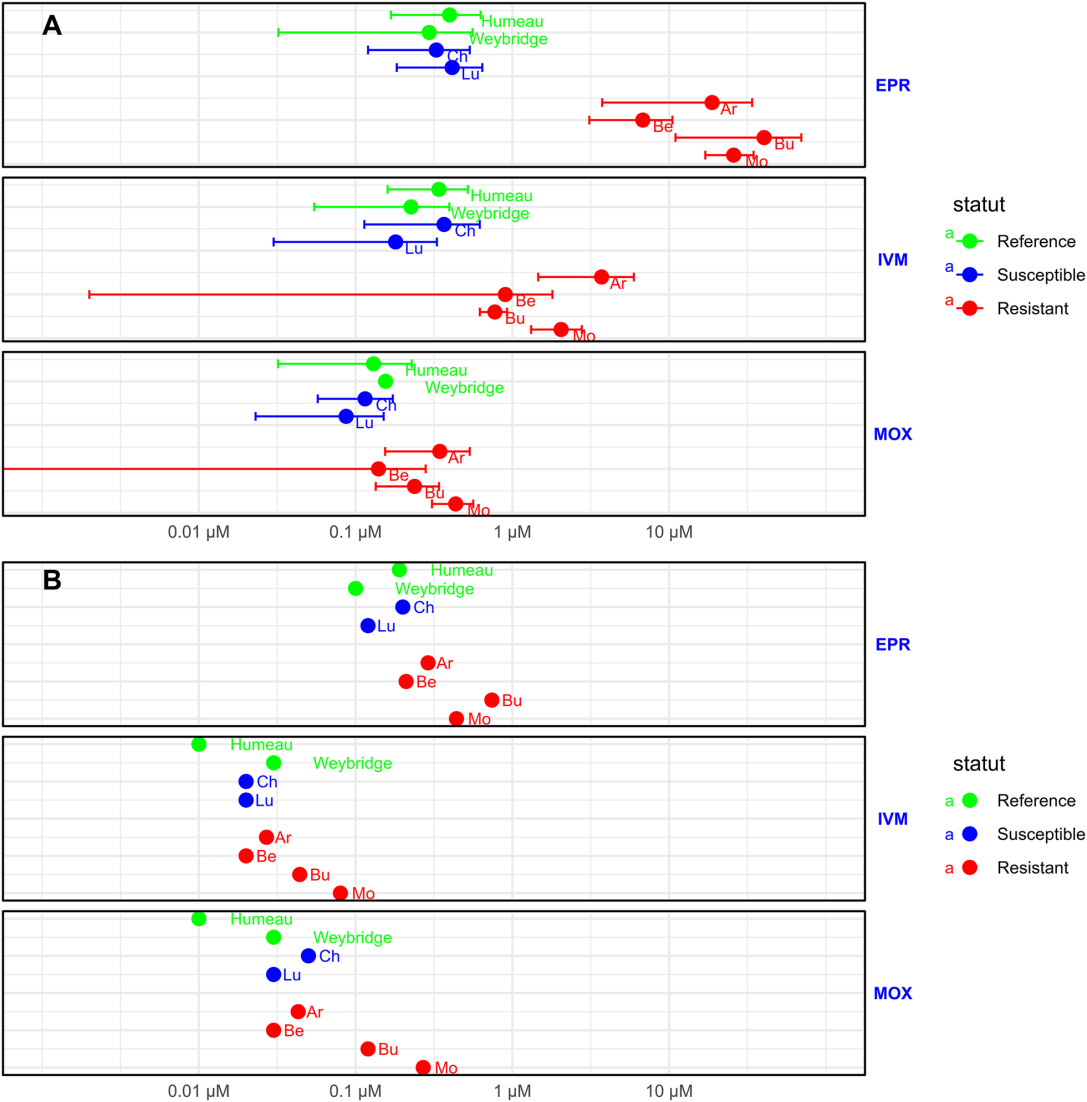
**ML potency of** ***H. contortus* isolates using motility tests with WMiT (A) and LDA (B).** The IC_50_ values were determined for EPR, IVM and MOX via the WMiT motility assay (**A**) and LDA (**B**). The four isolates issued from farms with clinical EPR treatment failure based on the FECRT are in red, the two isolates issued from farms with EPR efficacy are in blue, and the two reference EPR-susceptible isolates (Humeau and Weybridge reference strains) are in green. For WMiT, the values are the means ± SDs of 2–10 independent experiments.

The data obtained from the WMiT assay enabled a clear distinction between resistant and susceptible isolates on the basis of EPR IC_50_ values. In contrast, the IC_50_ values were less effective in distinguishing these groups for IVM and MOX. However, despite their significantly lower resistance factors, the EPR-resistant isolates still presented higher IC_50_ values for IVM and MOX than did the susceptible isolates did (Figure 2), but the confidence interval still overlapped between the EPR-susceptible and EPR-resistant isolates for MOX and IVM.

### Comparison of IC_50_ values obtained with motility using WMiT and LDA

Comparing the drug potency determined with WMiT (Figure 2A) and LDA (Figure 2B), we observed that the four susceptible isolates had IC_50_ values of similar magnitudes for all the MLs (Additional file 2). However, slight differences were observed, with higher IC_50_ values obtained with WMiT, especially for IVM (Humeau and Ch isolates) and MOX (Humeau and Weybridge isolates).

In contrast, notable differences emerged for resistant isolates. Interestingly, a sharp increase in the IC_50_ for EPR was observed with WMiT, whereas LDA showed a smaller difference in the IC_50_ between susceptible and resistant isolates. For IVM and MOX, increased IC_50_ values were observed in resistant isolates with both assays, although these variations were more pronounced with WMiT and depended on the isolate and the substance (Figure 2).

## Discussion

We successfully distinguished between EPR-resistant and EPR-susceptible *H. contortus* isolates from field samples using motility assays of L3 larvae, relating the drug potency values with the FECRT results. We confirmed here that the motility assay using WMiT is a robust and reproducible phenotype assay for evaluating EPR potency against *H. contortus* [22]. We applied this recent development to assess and compare the potency of ML on several *H. contortus* isolates collected from sheep dairy farms in a region of France with endemic parasite challenges.

In total, we collected *H. contortus* isolates from four farms where EPR treatment failed, as well as from two nearby farms where EPR was effective. *H. contortus* isolates were purified for other studies and used for phenotype characterization. The results were compared with data obtained with AH-susceptible reference strains used in previous studies, which have never been exposed to ML. Interestingly, the results obtained for EPR, MOX and IVM potency for these two susceptible strains align with those previously reported for MOX and IVM [34, 35, 40], suggesting that the differences in experimental conditions, including the number of L3 per well and the recording period after incubation, do not affect the consistency of our results. Purified isolates were used for this first step in validating the protocol, but the next step was to evaluate the protocol with non-purified *H. contortus* isolates and mixed species isolates.

Since LEV is a typical paralyzing agent, we assessed its effects on worm motility using WMicroTracker™ One (WMiT). The IC_50_ of LEV ranged from 8.6 to 47.5 µM, which is consistent with previous data reported for *H. contortus* [35, 37]. The FECRT status is not available for LEV, as its use is forbidden for dairy ewes. LDA exhibited no IC_50_ differences between the eight isolates, and they had never been exposed to LEV. Interestingly, under our experimental conditions, the sharp decrease in the sigmoid function reflects a narrow concentration window that induces a very rapid drug effect.

Humeau, Ch, and Lu exhibited similar susceptibility profiles to all three tested ML drugs, with MOX showing a slightly greater potency than IVM and EPR. In contrast, Weybridge is similarly susceptible to both MOX and IVM, but the EPR effect is less potent. This variation may reflect regional differences in drug response patterns or genetic factors among the worm populations, as the Weybridge strain originates from the UK, which is distant from southwestern France, where the other strains were collected. This is also the reason that the Humeau strain was selected as a reference for the calculation of RF.

The four isolates, Ar, Be, Bu, and Mo, presented exceptionally high IC_50_ values in larval motility assays using WMiT in response to EPR. Compared with the IC_50_ values for EPR in susceptible isolates, these values indicate the significantly reduced potency of the drug. Furthermore, the isolates were generated from coprocultures performed after EPR treatment, which may have reinforced the resistance selection. However, Alberich et al. conducted the same protocol on one non-purified resistant isolate with similar results [22]. The next step would be to test the isolate before any treatment on the farm. Finally, these results align perfectly with diagnostic findings from farms where the isolates were collected [1]. Notably, the resistance factors observed for EPR in these isolates underscore the precise discrimination between susceptible and resistant strains. Overall, these findings strongly highlight the robust resistance phenotype of these parasites, which is directly correlated with the treatment failures reported on farms.

Interestingly, LDA confirmed the ML-susceptible and ML-resistant phenotypes for most of the tested isolates. However, the EPR RFs obtained via LDA are all below 5, which is far lower than those obtained via WMiT. Conversely, the RFs obtained for MOX and IVM were consistent between LDA and WMiT for all the isolates. This suggests that LDA lacks sensitivity in detecting only the EPR resistance. LDA and WMiT differ according to the parasite stage they assess. While LDA evaluates the evolution from L1 to L3 across all three larval stages, WMiT focuses on L3 in a short time period [14, 23]. This raises the possibility that L1 or L2 might be more sensitive to the EPR than the other ML methods are, potentially explaining the differences in the results. However, others have used the microagar version of the larval development assay with resistant *H. contortus,* and they obtained resistance factors above ten for EPR [41], supporting differences between isolates.

Currently, in vivo and in vitro diagnostic methods are challenging to implement and do not allow for early detection of resistance [12, 42]. The advantage of using a larval motility assay to distinguish between sensitive and resistant *H. contortus* isolates is that it can be standardized. Using WMiT, larval motility is measured objectively by the device, reducing the subjectivity that can arise when it is assessed manually [22, 43]. Our results demonstrate that this method allows easy differentiation between *H. contortus* resistant or susceptible to EPR. Alberich et al. have also shown that such a methodology offers good repeatability and reproducibility [22].

We performed our test on exsheathed larvae according to previous protocols [23, 35, 37], providing more reproducible data than sheathed larvae (Petermann, unpublished data). This finding is in line with previous motility studies conducted on sheathed larvae, which suggested that motility may not be a reliable phenotype for distinguishing between susceptible and resistant isolates [16]. Interestingly, when resistance status is assessed through larval migration, as seen in different larval migration assays, compelling results are observed even with sheathed larvae [44, 45]. It might be valuable to further investigate the mechanisms underlying these two modalities—motility and mobility—which, while closely related, appear to exhibit some important differences.

Although our main interest in this study was *H. contortus*, larval motility can also be monitored in other species, such as *Cooperia oncophora*, *Ostertagia ostertagi* and *Teladorsagia circumcincta* [37]. The next steps will be, first, to test the dilution of resistant isolates with sensitive ones to determine the minimum resistance level detectable by the motility test and, second, to adapt this protocol to isolates of other strongyle species resistant to ML. Finally, the objective was to evaluate the protocol with mixed species before any treatment. Additionally, this method enables simultaneous testing of multiple substances. Diagnosing resistance by measuring larval motility could thus be applied to all the substances that cause paralysis in worms, including ML, LEV and monepantel. These substances are also used as positive controls in screening for drug repurposing [35, 36, 38]. Compared with LDA, measuring larval motility in L3s has the advantage of being more flexible in delays, which requires rapid sample submission and faeces containing nonevolved eggs. The L3 stage of the parasite can survive in this form for several weeks. For example, Chylinski et al. reported that *H. contortus* preserved at 4 °C for seven months maintained its fitness [44].

Preanalytical stages such as sample transmission are therefore easier and less time-dependent than larval development tests are [14, 38, 47]. This protocol also requires only a moderate number of larvae: less than 15 000 L3 is sufficient to test four different substances. Such an amount can be obtained from coproculture without the need to amplify the isolate if egg shedding is sufficient and coproculture yields are good. This technique is also quick; the protocol takes only 1.5 days to complete once L3 are available, which is far less than the FECRT, for example. Nevertheless, the method still needs optimization for direct use in field diagnostics. From the perspective of a diagnostic test at the farm level, the longest step is obtaining L3 through coproculture, which can be achieved by Taking samples approximately 15 days before the planned date of treatment, allowing farmers and veterinarians to determine the most effective substance. This would be similar to the time required for other L3-based tests, such as LMIA [45]. Currently, since all the tests have been conducted on purified *H. contortus* isolates, testing larval motility with WMiT on a larger number of field isolates from different regions would be an obvious step.

To further enhance the tool and provide field-accessible diagnostics, establishing threshold concentrations for each substance would be valuable. This would enable results to be reported to farmers in clear terms: susceptible, resistant or undefined. This is part of the main goal, which is to provide farmers with a definitive answer within 15 days so that they know which anthelmintic to use for treatment. Simplifying the diagnostic process would also enable surveys on the prevalence of resistance and help assess risk factors for the emergence of anthelmintic-resistant parasite populations. This, in turn, would allow for more precise recommendations to breeders, helping to delay the onset of resistance on their farms.

This study documents the presence of *H. contortus* isolates highly resistant to the EPR programme on dairy sheep farms. Strikingly, these resistant isolates were all collected from farms in geographically close areas, suggesting a potential resistance cluster. Our approach combines multiple robust diagnostic methods, establishing a sensitive protocol for assessing EPR potency by measuring larval motility with WMiT on farm-collected and purified *H. contortus*. The conclusion obtained with WMiT data was consistent with the results of the FECRT and the observations of on-farm treatment failures.

The automated method used to assess larval motility is rapid and standardized, delivering reliable EPR resistance detection in *H. contortus* isolates within a single day (once larvae are available), and only moderate larval numbers are used. Importantly, it has broad application potential for assessing resistance in other nematode species and against various anthelmintics, including ML, LEV, and monepantel.

In addition to highlighting the gravity of EPR resistance, particularly given that some of these isolates present increased resistance factors to other MLs, this study underscores the need for early, reliable diagnostics and increased surveillance. Effective resistance management allows farmers to select appropriate treatments and refine methods to delay resistance onset, enhancing the sustainability of livestock production. This integrated approach not only optimizes the use of existing drugs but also supports sustainable farming practices that can mitigate resistance, secure animal health, and sustain productivity in the face of evolving parasitic challenges.

## Supplementary Information

**Additional file 1. Concentration‒response curves for levamisole of the eight** ***H. contortus* isolates: Humeau, Weybridge, Ch, Lu, Be, Bu, Ar, and Mo. Each curve represents one experiment.**

**Additional file 2.** **IC**_**50**_** values and 95% confidence intervalsobtained with LDA for EPR, IVM and MOX.**

## Data Availability

The datasets used and/or analysed during the current study are available from the corresponding author upon reasonable request.
