# Supplementary figures and images for: Automated larval motility assays reveal links between eprinomectin treatment failure and drug resistance in Haemonchus contortus
Source: Vet Res. 2025 Oct 2;56:187. doi: 10.1186/s13567-025-01622-9 (PMC12492879; doi:10.1186/s13567-025-01622-9)

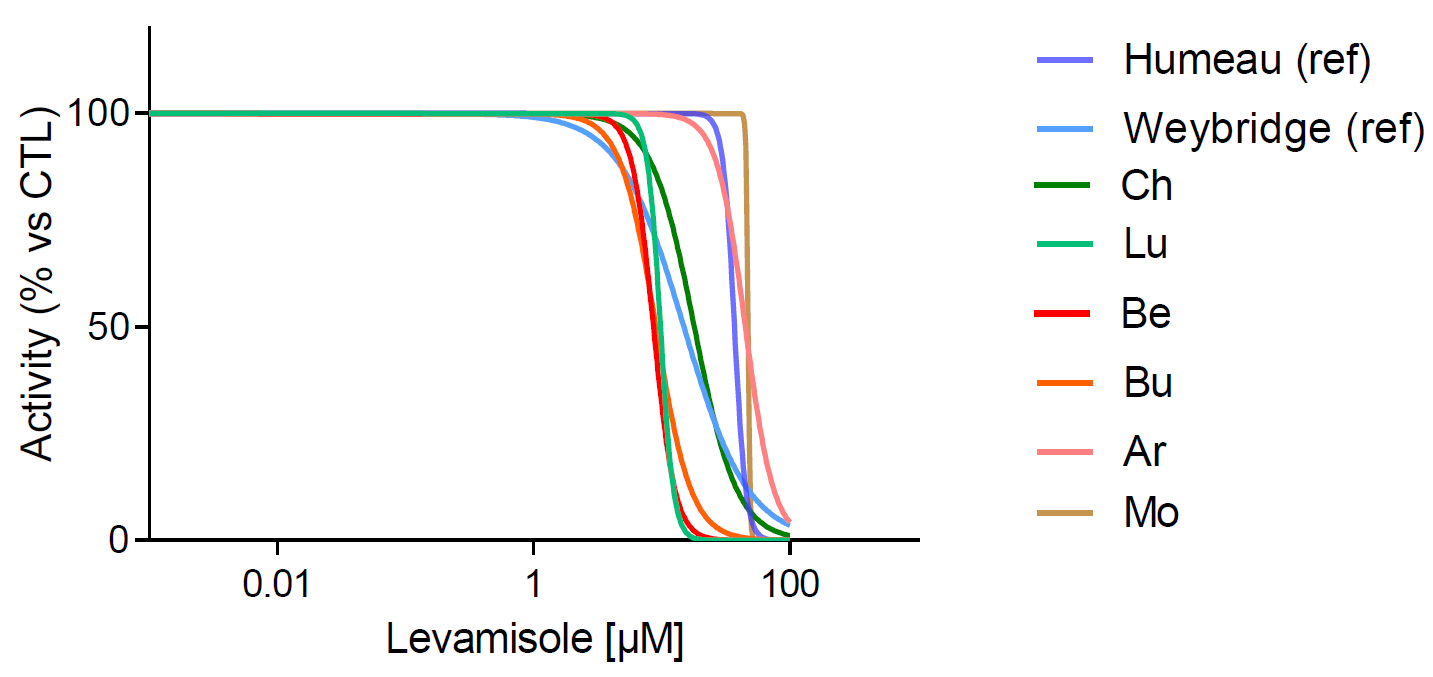

Supplement: Supplementary file 1 — Additional file 1. Concentration‒response curves for levamisole of the eight H. contortus isolates: Humeau, Weybridge, Ch, Lu, Be, Bu, Ar, and Mo. Each curve represents one experiment. [file 13567_2025_1622_MOESM1_ESM.png]

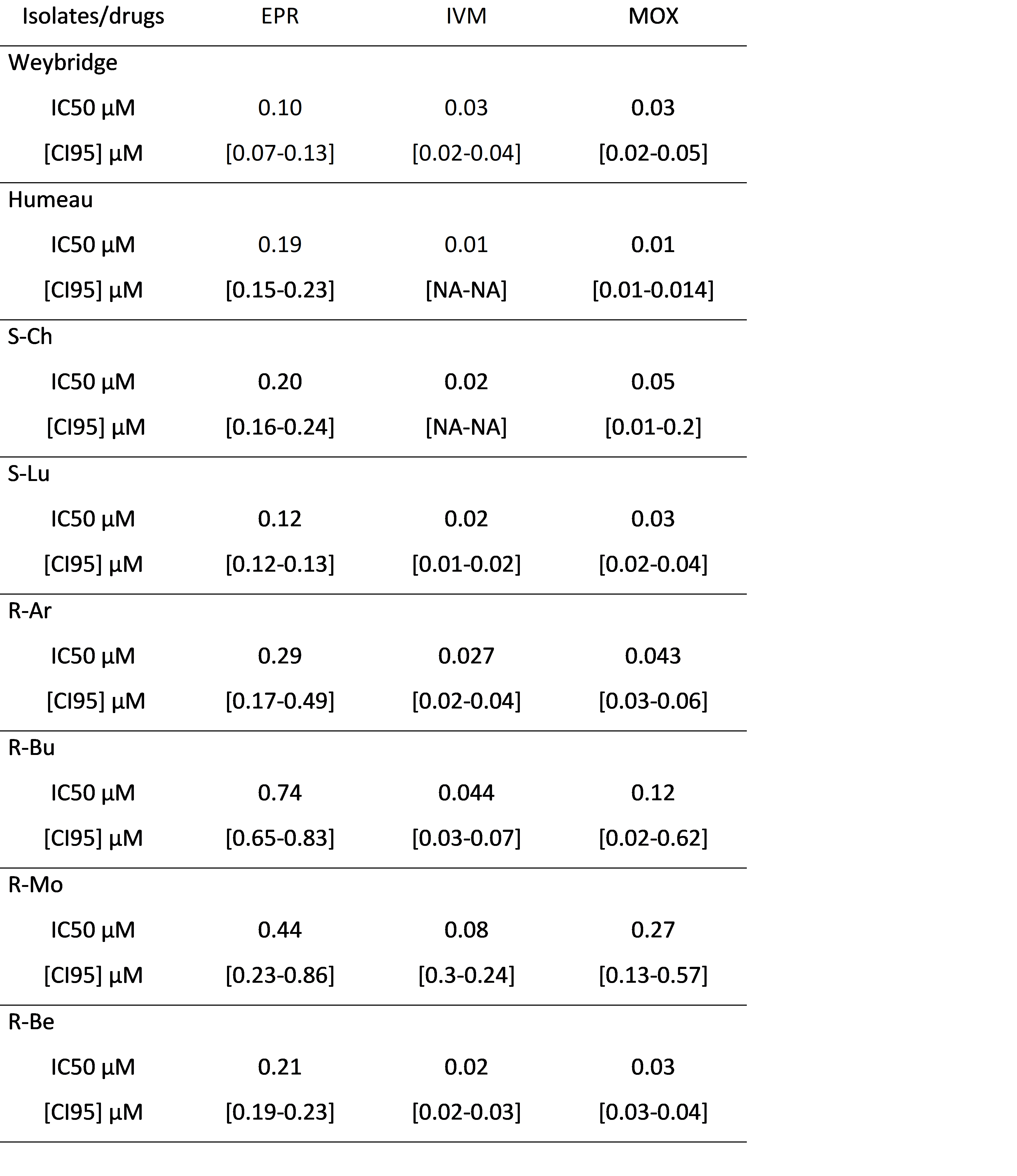

Supplement: Supplementary file 2 — Additional file 2. IC50 values and 95% confidence intervalsobtained with LDA for EPR, IVM and MOX. [file 13567_2025_1622_MOESM2_ESM.png]
